# Aminated Rapeseed Husks (*Brassica napus*) as an Effective Sorbent for Removing Anionic Dyes from Aqueous Solutions

**DOI:** 10.3390/molecules29040843

**Published:** 2024-02-14

**Authors:** Tomasz Jóźwiak, Urszula Filipkowska

**Affiliations:** Department of Environmental Engineering, University of Warmia and Mazury in Olsztyn, Warszawska St. 117a, 10-957 Olsztyn, Poland; urszula.filipkowska@uwm.edu.pl

**Keywords:** rapeseed husks, hulls, amination, cationization, epichlorohydrin, unconventional sorbent, sorption, dyes, Reactive Black 5, Reactive Yellow 84

## Abstract

The study investigated the effect of modifying rapeseed husks with ammonia and epichlorohydrin on their sorption capacity against anionic reactive dyes: Reactive Black 5 (RB5) and Reactive Yellow 84 (RY84). Its scope included sorbents characterization (FTIR, pH_PZC_), determination of pH influence on the sorption effectiveness of dyes, the adsorption kinetics of dyes, as well as the maximum sorption capacity. The study proved that the reaction of rapeseed husk biomass with ammonia can lead to its amination, namely to the introduction of amine functional groups into the material’s structure. The sorption effectiveness of RB5 and RY84 on the tested sorbents was the highest in the pH range of 2–3. The dye sorption kinetics was well described by the pseudo-second-order model. The sorption equilibrium time ranged from 90 to 180 min, and depended on the initial concentration of dyes and the number of amino groups on the sorbent’s surface. The most efficient of the sorbents tested were rapeseed husks pre-activated with epichlorohydrin and then aminated with ammonia. Their sorption capacity determined for RB5 and RY84 was 135.83 mg/g and 114.23 mg/g, respectively, which was 794% and 737% higher than that of the non-modified husks.

## 1. Introduction

Post-production wastewater from the textile, tanning, and paper industries can contain significant amounts of dyes, which pose especially severe threat to the natural environment. Their adverse effects afflict mainly the aquatic ecosystems.

Natural waters contaminated with dyes have limited transparency, which results in the inhibition of photosynthesis carried out by autotrophic aquatic organisms [1]. A significant proportion of the dyes are toxic to aquatic organisms [2], which, combined with the reduced oxygen content in the water reservoir, may diminish ecosystem’s biodiversity. In extreme cases, dye pollution causes significant degradation of the biosphere [3]. The perspective of the environment being destroyed by dyes prompts efforts to deploy the most effective methods for colored wastewater decolorization.

One of the simplest and most effective methods for removing dyes from post-production water is adsorption [4], which involves binding dye molecules on the sorbent’s surface. The most popular sorbents today include activated carbons [5], which consist mainly of elemental carbon, the characteristic feature of which is a very extensive surface area, exceeding 500 m^2^/g [6]. These sorbents have been proved effective in removing most industrial dyes [7]. Spent activated carbons can be regenerated several times [8]; however, their significant drawback is the high cost of their production and regeneration and, consequently, their high market price [9]. Hence, cheaper alternatives to activated carbons are sought currently that would increase the cost-effectiveness of wastewater decolorization through sorption.

An interesting material that can be used in this respect is chitosan, which is a polysaccharide and a deacetylated derivative of chitin, which is in turn the main constituent of arthropod exoskeletons. A properly prepared chitosan sorbent can be much more effective than the commercial-activated carbon [10]. In the case of anionic dyes, which are the most common type of colored wastewater pollutants, the sorption capacity of chitosan in the form of flakes may exceed 400 mg/g [11]. The high sorption properties of this material are due to the presence of many primary amine functional groups in its structure. Capable of easy protonation amine groups represent important sorption centers for anionic dyes (acidic and reactive ones) [12]. Chitosan can be sourced in industrial quantities from crab or shrimp exoskeletons, which are waste from the seafood processing industry [13]; however, the low availability of these raw materials in countries where there is no fishing for marine crustaceans is a huge disadvantage of chitosan to be used as a sorbent. In addition, market reserves of chitin and chitosan are depleted by medical companies which purchase them to produce, among others, dietary supplements, cosmetics, or dressing materials [14]. 

Given a similar chemical structure, alternatives to chitosan sorbents are sought among materials rich in plant polysaccharides, such as cellulose or hemicellulose. Raw materials based on plant biomass are very cheap and, unlike chitin and chitosan, widely available in most countries of the world [15]. However, high-cellulose materials, such as wood sawdust [16] or seed husks [17], exhibit relatively low sorption capacity towards anionic dyes (*Q_max_* < 15 mg/g). This is due to lignocellulosic plant biomass having a low number of cationic functional groups that are responsible for binding substances with a negative charge.

The problem of low sorption effectiveness of plant biomass regarding anionic dyes can be solved via its chemical modification, enriching its structure with cationic functional groups. One method used to cationize sorbents is their amination [18], which involves the material’s reaction with an aminating agent, as a result of which the sorbent gains amine groups. Ammonia is the most popular aminating agent. However, the plant biomass amination upon the direct reaction of ammonia with the polysaccharides and lignin contained in the sorbent is deemed to be little effective. In order to increase its effectiveness, the material may be pre-activated by, e.g., reaction with epichlorohydrin [19]. It is assumed that plant biomass enriched in amine functional groups can gain high sorption capacity towards anionic dyes.

The plant material used as a base to produce the amine sorbent should meet the conditions of low price, wide availability, and high content of polysaccharides (cellulose, hemicellulose) and lignin. An example of plant biomass meeting these conditions is rapeseed husks. Rape (*Brassica napus* L.) is one of the most popular crops. In 2020, its global production exceeded 70 million tons [20]. The husks account for approximately 20% of rape seed weight [21] and can be easily isolated from rapeseed oil cake, which is a waste product after oil pressing. The total content of polysaccharides and lignin accounts for about 60% of the husk biomass [22,23], which may indicate its potentially high susceptibility to chemical modification.

The study examined the influence of direct amination, and also amination with pre-activation by epichlorohydrin of the rapeseed husks on their sorption properties towards Reactive Black 5 and Reactive Yellow 84 anionic dyes.

## 2. Results and Discussion

### 2.1. Characteristics of Sorbents Tested (FTIR Analysis and C/N Elemental Analysis)

**Figure 1:**
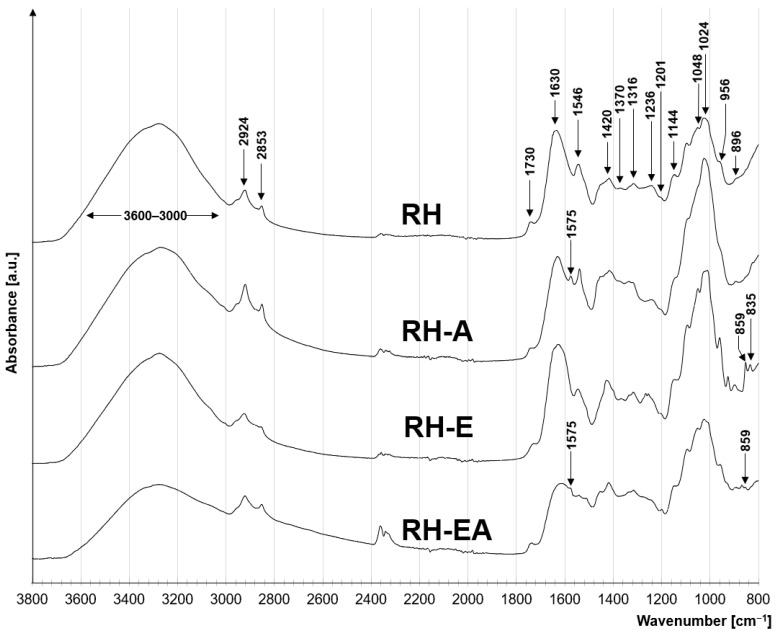
FTIR spectra for RH, RH-E, RH-A, and RH-EA.

The FTIR spectra of rapeseed husks suggests that they contain polysaccharides, lignins, and proteins (Figure 1). Polysaccharide-specific peaks can be found in the range of 1400–900 cm^−1^ [24]. The presence of C-O-C glycosidic bonds typical of saccharide rings is indicated by peaks visible at 1201 cm^−1^, 1144 cm^−1^, 1048 cm^−1^, and 1024 cm^−1^ [25]. The absorption band at 896 cm^−1^ indicate the stretching of the aromatic ring in the polysaccharide structure [26]. However, peaks at 1420 cm^−1^ and 1371 cm^−1^ are attributed to the tensile and bending vibrations of the -CH_2_ bonds of cellulose [27]. The peak at 1236 cm^−1^ is likely to indicate the C=C binding of the guaiacol ring, suggesting the presence of lignin. The lignin content in the rapeseed husks can also be indicated by a peak at 1316 cm^−1^ ascribed to the stretching of the C-O bond of the syringyl ring [28]. A wide absorption band of 3500–3000 cm^−1^ is attributed to the stretching of the O-H bond, typical of the hydroxyl functional groups found in both cellulose and lignin. The presence of proteins in the tested material can be indicated by peaks at 3278 cm^−1^, 1546 cm^−1^, and 1266 cm^−1^, which are attributed to the symmetrical stretching of the N-H bond (Amide A) and the N-H bonds of secondary and tertiary amides, respectively [29,30]. The peak at 1630 cm^−1^ can be attributed to the presence of carboxylic functional groups in the sorbent’s structure. Peaks at 2924 cm^−1^ and 2853 cm^−1^ can be attributed to asymmetric and symmetrical stretching vibrations of the -CH_2_- groups, which may derive from the lipid chains [31]. A peak at 1730 cm^−1^ indicates the presence of a carbonyl group C=O, which may be part of pectins and fatty acids of the tested material. 

The FTIR spectra of RH-A and RH-EA, i.e., aminated materials, show a peak at 1575 cm^−1^, which can be attributed to the C-N bond (Figure 1). The appearance of a new bond between carbon and nitrogen confirms that that the amine group attached to the sorbent’s structure [32].

In turn, the RH-E spectrum possesses characteristic peaks at 835 cm^−1^ and 859 cm^−1^, pointing to the presence of epoxide rings in the material [25] (Figure 1). This confirms the reaction of rapeseed husk biomass with epichlorohydrin. The peak visible at 850 cm^−1^ was also present in the RH-EA spectrum, which may suggest that not all epoxide groups were involved in material amination.

The elemental analysis showed that both the percentage of nitrogen and the N/C ratio of the sorbents tested increased in the following order RH-E < RH < RH-A < RH-EA (Table 1). The nitrogen content of RH-A and RH-EA biomass was 2.2% and 7.5% higher than in RH, respectively. This confirms the preliminary assumption that the amination of rapeseed husks is much more effective after biomass pre-activation with epichlorohydrin.

### 2.2. The Effect of pH on Dye Sorption Effectiveness

**Figure 2:**
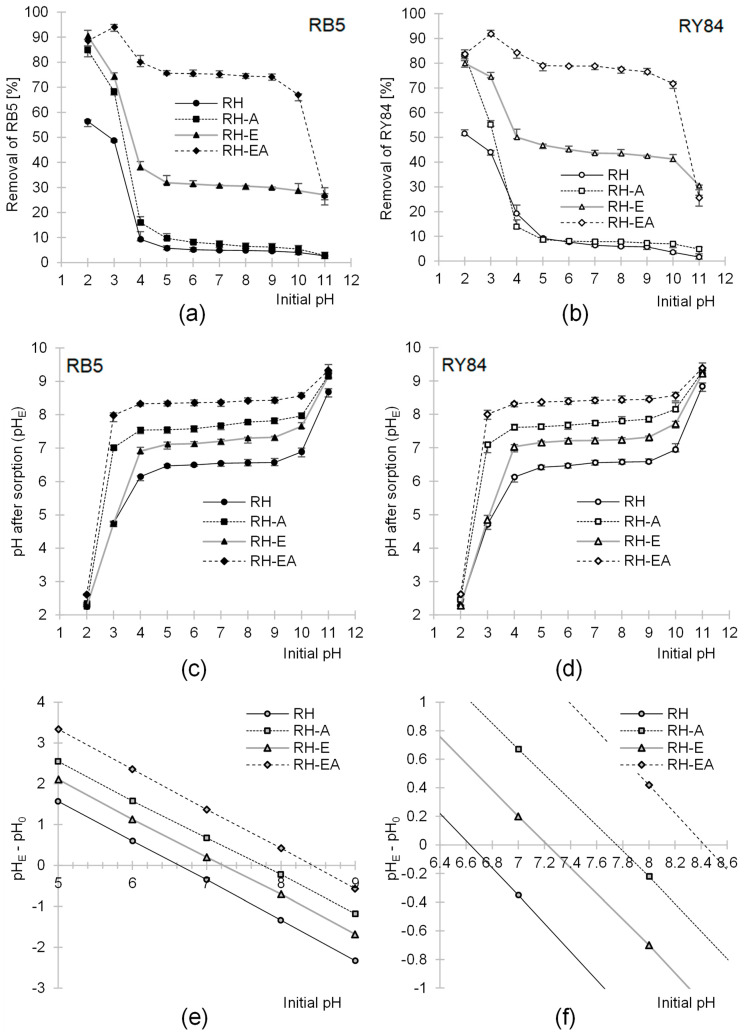
Effect of pH on sorption effectiveness of (**a**) RB5 and (**b**) RY84 on: RH, RH-A, RH-E, and RH-EA (average + range). Effect of sorbents on changes in solution pH during sorption of (**c**) RB5 and (**d**) RY84. (**e**,**f**) Determination of pH_PZC_ of the tested sorbents with the “drift” method. Sorbent dose—5 g/L, temp. 25 °C.

The effectiveness of dye sorption onto RH, RH-A, and RH-E was the highest at pH = 2 and onto RH-EA at pH = 3 (Figure 2a,b). In general, as the pH increased, the dye binding effectiveness on the sorbents tested decreased until the worst result had been achieved at pH = 11. However, the effect of pH on the RB5 and RY84 sorption effectiveness was not linear. The greatest decrease in the binding effectiveness of dyes onto RH, RH-A, and RH-E was recorded in the pH range of 3–4 and onto RH-EA in the pH range of 10–11. For each sorbent tested, however, the intensity of dye binding was similar in the pH range of 5–10. The sorption effectiveness of RH and RH-A in the pH range of 5–10 was low. RH-EA was the only sorbent tested that ensured a high sorption effectiveness in the discussed pH range (Figure 2a,b).

The high sorption effectiveness of anionic dyes at low pH resulted from the acquisition of a positive charge by the sorbent’s surface. In an acidic environment, with a significant excess of hydronium ions, the functional groups of sorbents were protonated. At pH < 6, most of the amine groups [33] underwent protonation, whereas also the hydroxyl groups started to undergo protonation presumably at pH < 3. 

In general, the low sorption effectiveness of anionic dyes at high pH was due to the acquisition of a negative charge by the sorbents, which might may have been caused by the deprotonation of certain functional groups, such as hydroxyl or carboxylic groups (derived from proteins).

The positive effect of low pH on the sorption of anionic dyes has also been observed in studies addressing the decolorization of aqueous solutions on other biomass-based unconventional sorbents, such as: the husk of sunflower seeds [34], pumpkin [35], cotton [36], and also activated carbons based on palm shells [37] or bamboo [38].

The slightly lower sorption effectiveness of RB5 and RY84 on RH-EA at pH = 2 compared to pH = 3 may have been due to the high competition of chlorides for sorbent’s active sites (-NH_3_^+^), and also to the deionization of anionic (sulfone) functional groups of dyes at pH = 2, which translated into weaker electrostatic interactions with dyes. The resulting effect is typical of most sorbents rich in amine functional groups [10,34,36]. 

A very large decrease in the sorption effectiveness of dyes on RH, RH-A, and RH-E, observed in the pH range of 3–4, was due to a relatively small number of basic functional groups (e.g., amine groups) on the surface of these sorbents. Unmodified rapeseed husks (RH), as a typically lignocellulosic material, had mainly hydroxyl functional groups, while the number of amine groups on their surface, derived mainly from proteins, was low. A similar number of amine groups was found for the rapeseed husks modified with epichlorohydrin (RH-E).

The husks modified directly with ammonia (RH-A) gained additional primary amine groups during the amination process. However, due to the low effectiveness of amination of the non-pre-activated biomass, the ratio of amine groups to hydroxyl groups on the RH-A surface was still relatively low. In conclusion, the main type of sorption centers on the surface of RH, RH-A, and RH-E were the -OH groups. Since at pH > 3, the hydroxyl groups were already predominantly in the non-ionized form, the electrostatic interaction between the dyes and the surface of RH, RH-A, and RH-E was weak, resulting in lower dye sorption effectiveness. This explains the significant decrease observed in sorption effectiveness of these sorbents in the pH range of 3–4 (Figure 2a,b). The higher sorption effectiveness of dyes onto RH-E in the pH range of 4–11 compared with RH and RH-A was probably due to the sorbent having epoxide functional groups acquired during the modification with epichlorohydrin. Thanks to these groups, RH-E could bind dyes also via chemisorption. 

The effectiveness of amination of rapeseed husks after their pre-activation with epichlorohydrin was much higher than in the case of non-pre-activated husks. For this reason, RH-EA had a substantially higher number of the amine functional groups than RH-A. The high number of easily-protonated amine groups translated into the high effectiveness of RB5 and RY84 sorption onto RH-EA in a broad pH range (Figure 2a,b).

The sorbents tested modified the pH of dye solutions during sorption (Figure 2c,d). For example, with the initial pH range of solutions being pH 5–10, the pH range of solutions after sorption was: pH 6.5–6.9 for RH; pH 7.5–8.0 for RH-A; pH 7.1–7.6 for RH-E; and pH 8.3–8.6 for RH-EA. These pH changes were due to the fact that the system always tends to reach the pH value that is close to the pH_PZC_ (the point of zero charge) of the sorbent. The pH_PZC_ values determined with the “drift” method reached pH_PZC_ = 6.6 for RH, pH_PZC_ = 7.75 for RH-A, pH_PZC_ = 7.20 for RH-E, and pH_PZC_ = 8.42 for RH-EA (Figure 2e,f). 

The pH_PZC_ determined for RH was below 7 (pH_PZC_ = 6.62), suggesting a slightly acidic nature of this sorbent, which might have been induced by a higher number of carboxylic groups than amino groups on the material’s surface. The pH_PZC_ = 7.2 of RH-E indicates its relatively neutral nature. The higher pH_PZC_ value determined for RH-E compared to RH may be due to a lower number of carboxylic groups on RH-E surface, which was probably caused by the reaction of these groups with epichlorohydrin during modification. Higher pH_PZC_ values of RH-A and RH-EA compared to those of RH and RH-E result from the incorporation of basic amine groups into the structure of sorbents during their amination. The much higher pH_PZC_ value of RH-EA than RH-A confirms that the effectiveness of amination of lignocellulosic biomass pre-activated with epichlorohydrin is much higher than that of the unmodified biomass.

Due to the fact that the colored wastewater rarely has a pH of <3, further stages of the study, described in Section 2.3 and Section 2.4, were carried out with solutions having pH = 3.

### 2.3. Dye Sorption Kinetics

**Figure 3:**
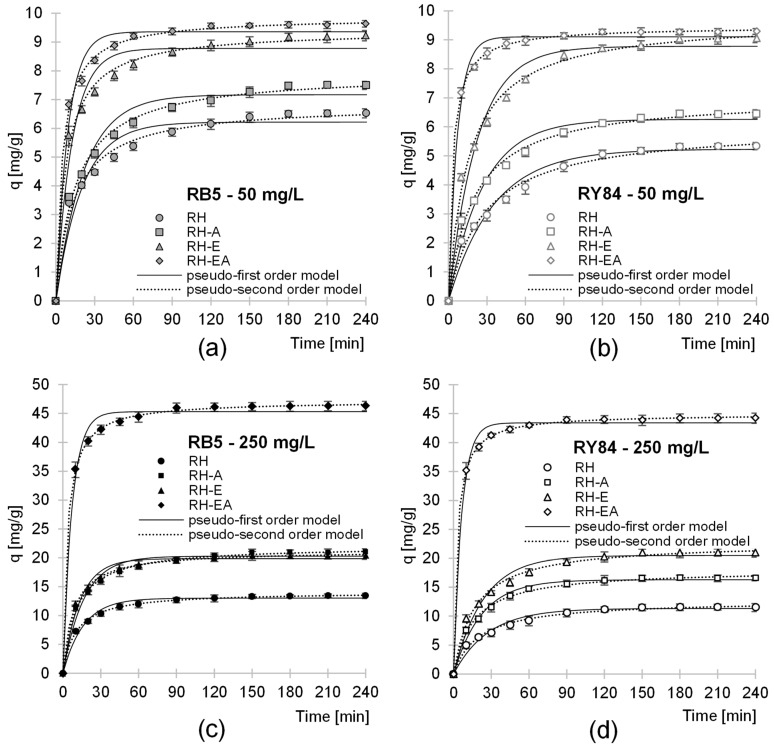
Sorption kinetics of: (**a**) RB5—50 mg/L, (**b**) RY84—50 mg/L, (**c**) RB5—250 mg/L, and (**d**) RY84—250 mg/L onto: RH, RH-A, RH-E, and RH-EA (average + range). The pseudo-first-order model and the pseudo-second-order model. Sorbent dose—5 g/L, pH 3, temp. 25 °C.

The time needed to reach the sorption equilibrium of RB5 and RY84 on the sorbents tested depended on the initial dye concentration and sorbent type. At the initial dye concentration of 50 mg/L, the sorption equilibrium was achieved on RH, RH-A, and RH-E after 180 min, whereas at dye concentration of 250 mg/L—after 150 min (Figure 3). Shorter equilibrium times were achieved in the analytical series with RH-EA, i.e., 120 min and 90 min at dye concentrations of 50 mg/L and 250 mg/L, respectively. The sorption of anionic dyes was the most intense at the beginning of the process. For example, after the first 10 min, the amount of RB5 sorbed onto RH, RH-A, RH-E, and RH-EA was: 52–55%, 48–54%, 59–63%, and 71–76% of *q_e_*, respectively.

Sorption equilibrium times similar to those for RH, RH-A, RH-E were also obtained in the studies on the sorption of RB5 on goldenrod biomass (150 min) [39], wheat straw (195 min) [40] and sunflower biomass (210 min) [41]. In turn, equilibrium times similar to that of RH-EA were recorded in the studies addressing the removal of RB5 on activated carbon from Carob tree (120 min) [42] as well as biochar from gasification of wood waste [43].

Shorter sorption times achieved in the analytical series with the higher initial concentrations of dyes might have been due to more frequent collisions of dye particles with active sites of the tested sorbents. Much shorter sorption times of dyes on RH-EA compared to RH, RH-A, and RH-E may have resulted from a very high number of protonated amine functional groups in the sorbent’s structure. The large total surface charge of RH-EA attracted electrostatically particles of anionic dyes from the solution, which increased the likelihood of sorbate collisions with sorption centers and accelerated their saturation.

Experimental data from studies into the RB5 and RY84 sorption kinetics onto RH, RH-A, RH-E, and RH-AE were described using pseudo-first and pseudo-second-order models (Figure 3, Table 2). In each analytical series, the pseudo-second-order model showed better fit to experimental data regardless of the sorbent type, dye, or its concentration, which is typical for the sorption of dyes on biosorbents. 

The values of the *k_2_* and *q_e_* constants determined from this model indicate a strong correlation between dye concentration in the solution and the intensity of its sorption. The constants determined from kinetic models suggest that the sorption rate of RB5 and RY84 on the tested sorbents increased in the RH < RH-A < RH-EA order. This can be explained by the increasing concentration of amine groups on the sorbent’s surface. The higher sorption rate of dyes onto RH-E than onto RH may be due to the fact that RH-E possesses epoxide groups, thanks to which it can bind dyes also via chemisorption, which further intensified RB5 and RY84 sorption in the present study.

**Figure 4:**
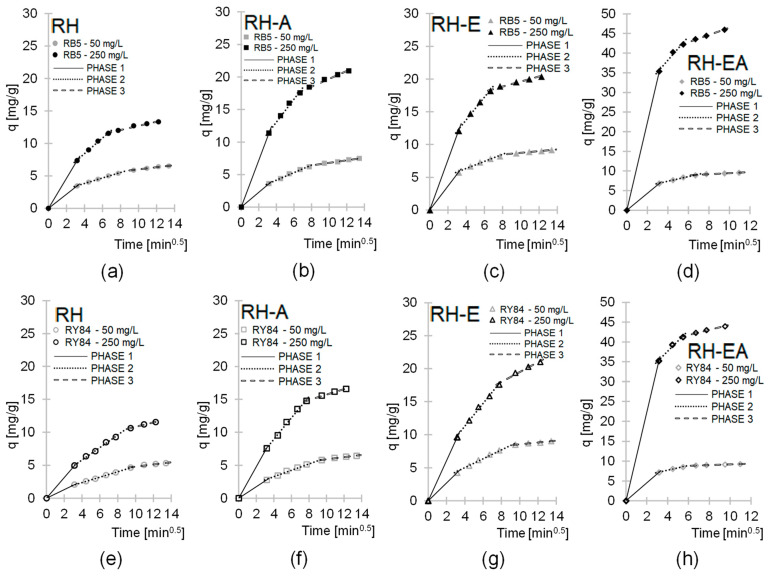
The intraparticle diffusion model of the sorption of RB5 onto (**a**) RH, (**b**) RH-A, (**c**) RH-E, (**d**) RH-EA, and RY84 onto (**e**) RH, (**f**) RH-A, (**g**) RH-E, and (**h**) RH-EA. Sorbent dose—5 g/L, pH 3, temp. 25 °C.

The sorption of dyes on the tested sorbents was also described by the intramolecular diffusion model (Figure 4, Table 3). Figures prepared based on this model indicate that RB5 and RY84 sorption onto RH, RH-A, RH-E, and RH-EA occurred in three phases, differing in intensity and duration. 

The values of *k_d1_*, *k_d2_*, and *k_d3_* constants determined from the intramolecular diffusion model increased in the RH < RH-A < RH-EA order, which confirms the positive effect of amination on the sorption capacity of rapeseed husks (Table 3). Analysis of the *k_d1_*, *k_d2_*, and *k_d3_* constants also confirms a strong correlation between dye concentration and its sorption effectiveness. 

The analytical series with RH-EA were characterized by much shorter duration of phase 2 and 3 compared to the series with RH, RH-E, and RH-A. Reaching adsorption equilibrium by the system within a shorter times span coupled with a large amount of dye that had been effectively bound are due to a very strong electrostatic interaction of the sorbent’s surface with dyes. 

The experimental series differing only in dye type were characterized by very similar values of *q_e_*, *k_d1_*, *k_d2_*, and *k_d3_* (determined from the pseudo-second-order model and the intramolecular diffusion model). This suggests that the sorption effectiveness of both dyes (RB5 and RY84) was similar under the same experimental conditions. This is probably due to the same chemical nature of dyes and the similar ratio of anionic (sulphone) functional groups to the molar mass of the entire dye.

### 2.4. Maximum Sorption Capacity

**Figure 5:**
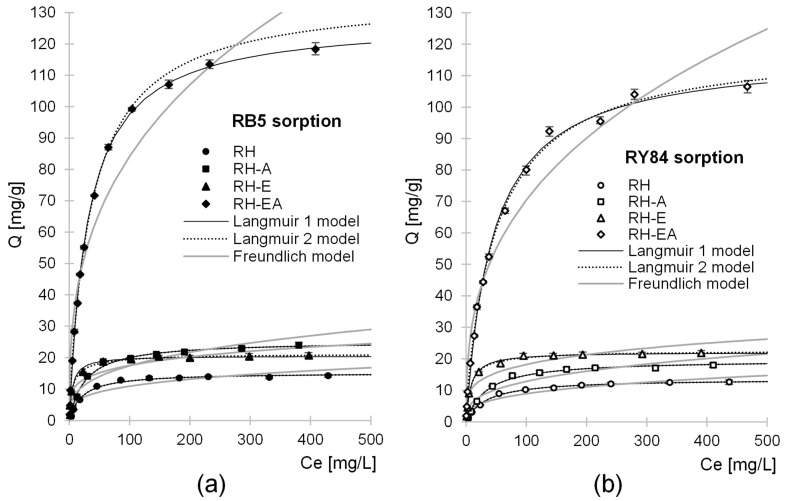
Isotherms of sorption of (**a**) RB5 and (**b**) RY84 onto: RH, RH-A, RH-E, and RH-EA (average + range). Langmuir 1, Langmuir 2, and Freundlich models. Sorbent dose—5 g/L, pH 3, temp. 25 °C.

Experimental data from studies on determining the maximum sorption capacity of the tested sorbents were described by means of Langmuir 1, Langmuir 2, and Freundlich sorption isotherms (Figure 5, Table 4). In all experimental series, the Langmuir 1 and 2 models showed better fit to experimental data compared to the Freundlich model. 

The values of *Q_max_* and *K_c_*/*K_1_*/*K_2_* constants determined from Langmuir 1 and Langmuir 2 models and the determination coefficients R^2^ were the same for RH and RH-A (Table 4), which indicates that only one type of sorption center—presumably the protonated hydroxyl functional groups—played a major role in the sorption of RB5 and RY84 on these sorbents. 

The sorption of dyes onto RH-E and RH-EA was better described with the Langmuir 2 than the Langmuir 1 model, which suggests that at least two different sorption centers, characterized by varying degrees of interaction with the sorbent, had a significant contribution to RB5 and RY84 binding. Presumably, ionized hydroxyl groups as well as epoxide groups served as these sorption sites for RH-E, whereas the protonated hydroxyl groups and ionized amino groups—for RH-E.

The maximum sorption capacity of RH reached 15.20 mg/g for RB5 and 13.65 mg/g for RY84 (Table 4). Compared to RH, better sorption effectiveness was demonstrated for RH-A, reaching 26.6 mg/g and 19.67 mg/g for RB5 and RY84, respectively. RH-E ensured similar sorption capacity to that of RH-A, as its *Q_max_* values were 21.24 mg RB5/g and 22.58 mg RY84/g. Among the analyzed sorbents, the highest sorption capacity of the tested anionic dyes was shown for RH-EA. Its sorption capacity determined for RB5 and RY84 reached 135.83 mg/g and 114.23 mg/g, respectively, and was 794% and 737% higher compared to RH.

As mentioned in the previous sections, the higher sorption capacity of the aminated rapeseed husks (RH-A, RH-EA) compared to the unmodified husks (RH) is due to the incorporation of amine functional groups into the sorbent’s structure during modification. Protonated amine groups strongly aided the sorption of anionic dyes. The significantly higher sorption capacity of RH-EA compared to RH-A is due to a higher number of amine functional groups on its surface. This confirms the assumption that the effectiveness of plant biomass amination is much higher after its pre-modification with epichlorohydrin. 

In turn, the higher sorption capacity of RH-E compared to RH is due to the presence of epoxide functional groups on its surface. Thanks to these functional groups, the physical adsorption of dyes on RH-E was aided by chemical adsorption. The sorption capacities obtained in the case of RB5 and RY84 were similar (Section 2.2), which may be due to the same chemical nature of these dyes and to the similar ratio of anionic functional groups to dye weight.

Relatively small *K_c_*/*K_1_* values (Table 4) determined for the tested sorbents from Langmuir models (1 and 2) may indicate a relatively small affinity of dyes to sorbents’ active sites. This means that the amount of dye bound is strongly correlated with its initial concentration. For this reason, the sorbents tested will exhibit the highest sorption capacity at high concentrations of dyes. The highest *K_c_*/*K_1_* values among the tested sorbents were determined for RH-E. This is due to the fact that it has many epoxide functional groups on its surface, which, unlike the ionized amino and hydroxyl groups, are able to produce stable covalent bonds with the sorbent.

Sorption capacities of the sorbent tested in this study were compared with those of other unconventional sorbents based on plant biomass as well as activated carbons (Table 5). The sorption effectiveness of rapeseed husks modified with epichlorohydrin and then aminated with ammonia (RH-EA) is much greater than that of non-modified lignocellulosic biosorbents, such as seed husks, plant stalks or sawdust (Table 5). The process of biomass amination that had earlier been pre-activated with epichlorohydrin effectively increases the sorption capacity of other lignocellulosic materials, like sunflower seeds and buckwheat sed husks, wheat straw or Canadian goldenrod biomass, as described in our previous works [19,34,39,40].

RH-EA does not match the performance of high-quality chitosan (DD = 90%), but has a higher sorption capacity than most sorbents based on activated carbon. In addition, its sorption effectiveness is comparable to that of chitin. This suggests the possibility of using RH-EA as an alternative to commercial sorbents.

Theoretically, the regeneration of sorbents spent during sorption (RH, RH-A, RH-E, and RH-EA) by desorption of dyes would be cost-ineffective. Such a process would require strong bases and would generate wastewater that would have to be managed. 

The most frequently used method for managing spent sorbents is to store them in landfills [55]. However, it should be remembered that the dyes bound with the “post-sorbent” may be washed out and contaminate local soils as well as ground and surface waters. Taking into account the fact that both plant biomass and dyes have a high heating value, a better solution would be to dry the post-sorbents and recover energy through their co-combustion, e.g., in a heating plant [56,57]. A drawback of combusting sorbents containing dyes is the possibility of emitting harmful substances into the atmosphere. However, the risk of air pollution can be minimized by using an appropriately high combustion temperature (850–1150 °C). Another possibility could be the carbonization and activation of spent sorbents, which would lead to the formation of full-value activated carbons [58]. Preliminary research conducted by the authors also suggests the feasibility of fermenting spent sorbents based on plant biomass to produce biogas.

## 3. Materials

### 3.1. Rapeseed Husks

Rapeseed husks (*Brassica napus*) were obtained from rapeseed oil cake provided by the rapeseed oil pressing plant operating in the Warmian–Masurian Voivodeship (Poland). The oil cake derived from industrial rapeseeds from the local harvest of 2020.

Prior to the study, the husks were pre-separated from the cake and purified, as described in more detail in Section 4.1. The content of husks in the cake was 31%. The composition of purified dry husks was as follows: cellulose—13.7%, hemicellulose—19.0%, lignin—25.0%, pectins—12.0%, proteins—18.1%, and other components (lipids, ash)—12.2% (determined by the HPLC method) [22,23].

### 3.2. Dyes

Industrial dyes used in the research (Reactive Black 5, Reactive Yellow 84) were provided by the dye producing plant Zakład Przemysłu Barwników “BORUTA-ZACHEM KOLOR SA” in Zgierz (Poland), and their most important parameters are collated in Table S1. 

### 3.3. Chemical Reagents

Hydrochloric acid (HCl, 37%) and sodium hydroxide (NaOH, granules, >99.9%) were used to correct the pH of dye solutions. Ammonia (NH_3_–H_2_O 25%) was used for amination of the sorbent material. Epichlorohydrin (C_3_H_5_ClO, >99.0%) was used for the modification and activation of the sorbent material. Acetone (C_3_H_6_O, >99.5%) was used to clean the diamond crystal in the ATR attachment of the spectrometer. Buffer solutions (pH 4 ± 0.05/pH 7 ± 0.05/pH 10 ± 0.05) were used to calibrate the pH meter. All chemical reagents used were purchased from POCH S.A., Gliwice, Poland, and were of at least p.a. (analytical) purity. 

### 3.4. Laboratory Equipment

The HI 221 pH-meter (Hanna Instruments, Woonsocket, RI, USA) was used to measure and correct the pH value of the solutions. The water bath shaker type 357 (Elpin-Plus, Lubawa, Poland) was used for the modification/activation of the rapeseed hulls with epichlorohydrin. The SK-71 laboratory shaker (JEIO TECH, Daejeon, Republic of Korea) and the MS-53M multi-channel stirrer (JEIO TECH, Daejeon, Republic of Korea) were used for the sorption process. The UV-3100 PC—UV/Visible spectrophotometer (VWR spectrophotometers, VWR International LLC., Mississauga, ON, Canada) was used to determine the dye concentration in the solutions. The FT/IR-4700LE FT-IR spectrometer with a single reflection ATR attachment (JASCO International, Tokyo, Japan) was used to obtain the FTIR spectra of the sorbent. The FLASH 2000 analyzer (Thermo Scientific, Waltham, MA, USA) was used to measure the carbon and nitrogen content in the sorbents.

## 4. Methods

### 4.1. Preparation of Rapeseed Husks (RH)

The rapeseed oil cake was placed in a beaker with hot deionized water (50–55 °C), and the contents of the beaker were stirred intensively for 5 min with a spatula. During the mixing, the crushed seed nuclei were separated from the husks. After mixing, the slow-floating rapeseed husks were separated on a laboratory screen with a mesh diameter of 0.5 mm. The crushed fragments of the nuclei passed through the screen together with the water. Rapeseed husks separated on the screen were moved into a beaker with hot deionized water, and the procedure of husk mixing and separation on the screen was repeated several times until the husks were completely purified of the remains of the crushed nuclei (the last cycle of husk purification was carried out when the water after washing the husks was clear, devoid of turbidity). Afterwards, the husks were dried at a temperature of 105 °C. The purified and dried rapeseed husks (RH) were ready for analyses.

### 4.2. Preparation of Aminated Rapeseed Husks (RH-A)

Rapeseed husks (RH) were weighed (100 g) into the beaker (vol. 500 mL), which was then filled with 400 mL of ammonia water (25%). The beaker was secured with a parafilm and placed on a magnetic stirrer (100 r.p.m) under the fume cupboard for 24 h. Afterwards, the modified husks were sieved through a laboratory screen and washed with a large volume of deionized water. The process of husk washing was continued until a neutral pH (pH < 7.5) was obtained in the filtrate and the characteristic odor of ammonia was lost. After drying at 105 °C, the ammonia-modified rapeseed husks (RH-A) were ready for analyses.

### 4.3. Preparation of Rapeseed Husks Modified with Epichlorohydrin (RH-E)

Rapeseed husks (RH) were weighed (100 g) into a conical flask (vol. 1000 mL). A 95% epichlorohydrin solution (400 mL) with pH = 12 was then added to the flask (pH of the epichlorohydrin solution was corrected by adding an appropriate amount of an aqueous NaOH solution to it). The flask was secured with a parafilm and inserted into a water bath (120 r.p.m., vibration amplitude of 30 mm, 60 °C) placed under the fume cupboard. After 24 h, the husks were separated from the solution on a laboratory screen and washed with distilled water until a neutral pH (pH < 7.5) was obtained in the filtrate and the odor of epichlorohydrin was no longer perceptible. The epichlorohydrin-modified husks (RH-E) were then dried (105 °C), and afterwards they were ready for analyses.

### 4.4. Preparation of Aminated Rapeseed Husks Pre-Activated with Epichlorohydrin (RH-EA)

The husks of rapeseed modified with epichlorohydrin (RH-E) (prepared as described in Section 4.3) were aminated (as described in Section 4.2), which resulted in a double-modified sorbent (RH-EA).

All sorbents (RH, RH-A, RH-E, RH-EA) were “dry” stored in sealed polyethylene containers at a temperature of 4 °C.

**Figure 6:**
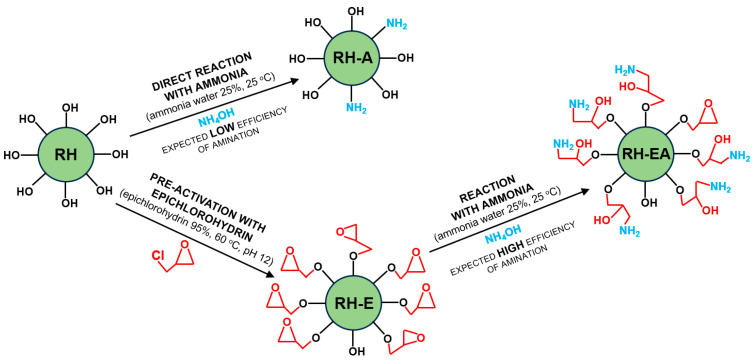
Scheme of preparation of RH, RH-A, RH-E, and RH-EA.

A simplified diagram of the preparation of RH-A, RH-E, and RH-EA is shown in Figure 6.

### 4.5. Analyses of pH Effect on Dye Sorption Effectiveness

The sorbent (0.5 g portions) was weighed into a series of 250 mL conical flasks. Dye solutions (100 mL) with a concentration of 100 mg/L and pH between 2 and 11 were then added to the flasks. Next, the flasks were placed on a multi-station laboratory shaker (150 r.p.m., vibration amplitude of 30 mm) and shaken for 120 min. Afterwards, samples of the solutions (10 mL) were collected from the flasks with an automatic pipette for the analysis of dye concentration. The pH value of the solutions was also measured after the sorption process.

### 4.6. Analyses of Dye Sorption Kinetics

Portions of the sorbent (5 g) were weighed into beakers (1000 mL), which were then filled with dye solutions (1000 mL) with concentrations of 50/250 mg/L. The pH of the solutions was determined based on analyses described in Section 4.5. The beakers were then placed on multi-station magnetic stirrers (200 r.p.m., Teflon-coated stirrer 50 × 8 mm). After the appointed times (0, 10, 20, 30, 45, 60, 90, 120, 150, 180, 210, 240, 300, 360 min), samples of the solutions (2 mL) were collected from the beakers with an automatic pipette to determine the concentration of the dye remaining in the solution.

### 4.7. Analyses of the Maximum Sorption Capacity of the Sorbents Used in the Study

Portions of sorbents (0.5 g) were weighed to a series of conical flasks. Then, 100 mL of dye solutions with the concentrations of 10–500 mg/L (for RH, RH-A, RH-E) or 10–1000 mg/L (for RH-EA) were poured into the flasks. The higher solution concentrations used in the experimental series with RH-EA were due to its much higher sorption capacities. The pH of the dye solutions was determined, as described in Section 4.5. The flasks were placed on a multi-station shaker (150 r.p.m., vibration amplitude of 30 mm) for the time of sorption equilibrium (determined based on analyses described in Section 4.6). Afterwards, samples of the solutions (10 mL) were collected from the flasks with an automatic pipette to analyze the concentration of the remaining dye.

### 4.8. Comments to Section 4.5, Section 4.6 and Section 4.7

Portions of sorbents added to flasks or beakers were prepared on a precision scale exact to 0.001 g. The mixing parameters set on the shaker or the multi-station stirrer ensured that the sorbent was mixed in the entire volume of the solution. The concentration of dyes remaining in the solution was determined with the spectrophotometric method on a UV-VIS spectrophotometer with a cuvette, at an optical path length of 10 mm. The measurements were performed at the wavelength maximum (λ_max_) of the dyes. The calibration curves plotted for RB5 and RY84 dyes allowed their concentration to be measured in the range of 0–50 mg/L. For higher concentrations, the solutions were diluted with deionized water. All experimental series were performed in three replicates. During the analyses, a constant temperature of 25 °C was maintained in the laboratory.

The pH_PZC_ of the sorbents was determined using the “drift” method [59].

### 4.9. FTIR Analysis of the Tested Sorbents

The infrared spectrum analysis of the tested materials was performed using an FT/IR-4700LE spectrometer with a single reflection diamond crystal ATR attachment (JASCO International, Tokyo, Japan). 

Before analysis, the materials were dehydrated using a hydraulic press. The scanning range of the samples covered the wavelength range from 4000 to 400 cm^−1^. The resolution of each spectrum was 1 cm^−1^. Each sample was measured 64 times, and the results were averaged. After each measurement, the ATR diamond crystal was cleaned with acetone and then wiped with a cellulose towel.

### 4.10. Elemental Analysis of the Tested Sorbents

The contents of carbon and nitrogen in the tested sorbents were determined using the Flash 2000 elemental analyzer, based on the method of dynamic, complete combustion of samples in a redox furnace with electronically controlled temperature. 

The analyzer worked in the C/N mode. The amount of an organic sample burned in each measurement ranged from 5 to 7 mg. The measurement range was 0.01–100.00%. The measurement sensitivity declared by the manufacturer was up to several ppm.

### 4.11. Data Analysis

The amount of dye adsorbed on the tested sorbents was determined from Formula (1).

(1) $$Q_{S}=\left(C_{0}-C_{S}\right)\times \frac{V}{m}$$

*Q_S_*—mass of sorbed dye [mg/g]
*C*_0_—initial concentration of dye [mg/L]
*C_S_*—concentration of dye after sorption [mg/L]
*V*—volume of the solution [L]
*m*—mass of the sorbent [g].

The kinetics of dye sorption onto the tested sorbents was described using the pseudo-first-order model (2), the pseudo-second-order model (3) [60], and the intramolecular diffusion model (4) [61].

(2) $$q=q_{e}\times \left(1-e^{\left(-k_{1}\times t\right)}\right)$$

(3) $$q=\frac{\left(k_{2}\times {q_{e}}^{2}\times t\right)}{\left(1+k_{2}\times q_{e}\times t\right)}$$

(4) $$q=k_{id}\times t^{0.5}$$

*q*—instantaneous value of the sorbed dye [mg/g]
*q_e_*—the amount of dye sorbed at the equilibrium state [mg/g]
*t*—time of sorption [min]
*k*_1_—pseudo-first-order adsorption rate constant [1/min]
*k*_2_—pseudo-second-order adsorption rate constant [g/(mg·min)]
*k_id_*—intramolecular diffusion model adsorption rate constant [mg/(g·min^0.5^)].

The experimental data obtained from studies on the maximum sorption capacity of the analyzed sorbents were described using three popular sorption models: the Langmuir 1 isotherm (5) [62], the Langmuir 2 isotherm (Langmuir double isotherm) (6) [63], and the Freundlich isotherm (7) [62].

(5) $$Q=\frac{\left(Q_{max}\times K_{C}\times C\right)}{\left(1+K_{C}\times C\right)}$$

(6) $$Q=\frac{\left(b_{1}\times K_{1}\times C\right)}{\left(1+K_{1}\times C\right)}+\frac{\left(b_{2}\times K_{2}\times C\right)}{\left(1+K_{2}\times C\right)}$$

(7) $$Q=K\times C^{\frac{1}{n}}$$

*Q*—mass of the sorbed dye [mg/g]
*Q_max_*–maximum sorption capacity in Langmuir equation [mg/g]
*b*_1_—maximum sorption capacity of sorbent (type I active sites) [mg/g]
*b*_2_—maximum sorption capacity of sorbent (type II active sites) [mg/g]
*K_C_*—constant in Langmuir equation [L/mg]
*K*_1_,*K*_2_—constants in Langmuir 2 equation [L/mg]
*K*—the equilibrium sorption constant in Freundlich model
*n*—Freundlich equilibrium constant
*C*—concentration of the dye remaining in the solution [mg/L].

## 5. Conclusions

The amination of rapeseed husks significantly improves their sorption capacity towards anionic dyes. The prerequisite for high effectiveness of sorbent amination is its pre-activation/pre-modification with epichlorohydrin.

The pH of the solutions had a strong impact on the sorption effectiveness of dyes. In general, the intensity of dye sorption on the tested sorbents decreased with pH increases in the system. Sorbents modified the pH of the solutions during sorption.

The sorption equilibrium time ranged from 90 to 180 min, and depended on the initial concentration of dyes and the number of amine groups on the sorbent’s surface. Generally, shorter sorption times were obtained in the series with higher concentrations of the dyes, and also in the case of sorbents with the highest content of -NH_2_ groups.

RB5 and RY84 sorption on the tested sorbents occurred in three main phases, differing in intensity and duration.

Only one type of sorption center played a major role in the sorption of dyes onto RH and RH-A, presumably ionized hydroxyl functional groups on the sorbent’s surface. In the case of RH-E and RH-EA, at least two types of active centers had a strong impact on their sorption, these being presumably ionized hydroxyl groups, epoxide groups of RH-E, as well as protonated hydroxyl groups and protonated amine groups of RH-EA.

## Figures and Tables

**Table 1:** Carbon and nitrogen content in tested sorbents. Analyses carried out on the elemental analyzer—FLASH 2000 (THERMO SCIENTIFIC, USA) (three repetitions of measurements).

Type of Sorbent	Carbon Content [%]	Nitrogen Content [%]	N/C Ratio
RH	42.51 ± 0.16	1.052 ± 0.010	0.0247
RH-A	42.25 ± 0.14	1.075 ± 0.008	0.0254
RH-E	43.48 ± 0.20	1.043 ± 0.004	0.0240
RH-EA	43.39 ± 0.18	1.131 ± 0.003	0.0261

**Table 2:** Kinetic parameters of sorption of RB5 and RY84 onto RH, RH-A, RH-E, and RH-EA, determined from the pseudo-first-order and pseudo-second-order models (based on the average of three measurements) + sorption equilibrium time.

Sorbent	Dye	Dye Conc.	Pseudo-First-Order Model			Pseudo-Second-Order Model			Exp. Data	Equil. Time
			*k_1_*	*q_e_*_, cal._	R^2^	*k_2_*	*q_e_*_, cal_.	R^2^	*q_e_*_, exp._	
		[mg/L]	[1/min]	[mg/g]	-	[g/mg·min]	[mg/g]	-	[mg/g]	[min]
RH	RB5	50	0.0495	6.22	0.9488	0.0108	6.84	0.9881	6.53	180
		250	0.0618	13.00	0.9783	0.0069	14.10	0.9985	13.44	150
	RY84	50	0.0286	5.23	0.9687	0.0060	6.03	0.9874	5.35	180
		250	0.0365	11.32	0.9709	0.0039	12.74	0.9912	11.54	150
RH-A	RB5	50	0.0459	7.17	0.9633	0.0084	7.93	0.9936	7.50	180
		250	0.0600	20.26	0.9717	0.0043	22.02	0.9975	21.05	150
	RY84	50	0.0371	6.25	0.9703	0.0072	7.04	0.9933	6.46	180
		250	0.0448	16.29	0.9849	0.0036	18.04	0.9956	16.63	150
RH-E	RB5	50	0.0792	8.78	0.9622	0.0140	9.42	0.9951	9.24	180
		250	0.0738	19.83	0.9810	0.0058	21.27	0.9988	20.44	150
	RY84	50	0.0446	8.77	0.9728	0.0067	9.71	0.9950	9.06	180
		250	0.0420	20.47	0.9713	0.0026	22.77	0.9942	21.02	150
RH-EA	RB5	50	0.1070	9.36	0.9796	0.0207	9.86	0.9982	9.64	120
		250	0.1370	45.30	0.9889	0.0062	47.18	0.9997	46.35	90
	RY84	50	0.1402	9.10	0.9903	0.0328	9.46	0.9998	9.30	120
		250	0.1537	43.39	0.9924	0.0081	44.91	0.9998	44.28	90

**Table 3:** Rate constants of RB5 and RY84 diffusion determined from a simplified intraparticle diffusion model. * [mg/(g·min^0.5^)].

Sorbent	Dye	Dye Conc.	Phase 1			Phase 2			Phase 3		
			*k_d1_* *	Dur. Time	R^2^	*k_d2_*	Dur. Time	R^2^	*k_d3_*	Dur. Time	R^2^
		[mg/L]	*	[min]	-	*	[min]	-	*	[min]	-
RH	RB5	50	1.0907	10	0.(9)	0.4250	50	0.9988	0.1648	120	0.9834
		250	2.3211	10	0.(9)	1.1989	35	0.9951	0.3184	105	0.9751
	RY84	50	0.6514	10	0.(9)	0.4097	80	0.9997	0.1670	90	0.9279
		250	1.5748	10	0.(9)	0.9419	50	0.9965	0.3311	90	0.9864
RH-A	RB5	50	1.1416	10	0.(9)	0.5767	50	0.9919	0.1947	120	0.9946
		250	3.5955	10	0.(9)	1.7621	35	0.9888	0.6045	105	0.9869
	RY84	50	0.8791	10	0.(9)	0.4828	80	0.9903	0.1624	90	0.9815
		250	2.3970	10	0.(9)	1.6056	50	0.9951	0.4099	90	0.9940
RH-E	RB5	50	1.8215	10	0.(9)	0.5362	50	0.9836	0.1296	120	0.9907
		250	3.8200	10	0.(9)	1.7271	35	0.9912	0.3822	105	0.9771
	RY84	50	1.3503	10	0.(9)	0.6725	80	0.9872	0.1367	90	0.9870
		250	3.0453	10	0.(9)	1.7225	50	0.9948	0.7566	90	0.9795
RH-EA	RB5	50	2.1598	10	0.(9)	0.5897	35	0.9892	0.1496	75	0.9364
		250	11.189	10	0.(9)	3.0029	20	0.9744	0.9229	60	0.9971
	RY84	50	2.2705	10	0.(9)	0.5945	20	0.9915	0.0951	90	0.9993
		250	11.140	10	0.(9)	2.6307	20	0.9858	0.6610	60	0.9887

**Table 4:** Constants determined from Langmuir 1, Langmuir 2, and Freundlich models.

Sorbent	Dye	Langmuir 1 Model			Langmuir 2 Model						Freundlich Model		
		*Q_max_*	*K_c_*	R^2^	*Q_max_*	*b_1_*	*K_1_*	*b_2_*	*K_2_*	R^2^	*k*	*n*	R^2^
		[mg/g]	[L/mg]	-	mg/g	mg/g	L/mg	mg/g	L/mg	-	-	-	-
RH	RB5	15.20	0.048	0.9916	15.20	7.60	0.048	7.60	0.048	0.9916	3.19	0.267	0.8506
	RY84	13.65	0.030	0.9974	13.65	6.77	0.030	6.88	0.030	0.9974	2.16	0.309	0.9162
RH-A	RB5	26.26	0.038	0.9926	26.26	12.63	0.038	12.63	0.038	0.9926	4.09	0.315	0.8918
	RY84	19.67	0.030	0.9935	19.67	9.83	0.030	9.84	0.03	0.9935	2.94	0.320	0.8870
RH-E	RB5	20.54	0.201	0.9891	21.24	15.70	0.303	5.54	0.032	0.9926	6.75	0.207	0.8746
	RY84	22.08	0.134	0.9948	22.58	20.35	0.153	2.23	0.014	0.9954	6.42	0.227	0.8712
RH-EA	RB5	127.72	0.032	0.9991	135.83	130.30	0.026	5.53	0.955	0.9993	17.15	0.346	0.9376
	RY84	117.23	0.023	0.9954	119.98	114.23	0.019	5.75	2.70	0.9978	13.71	0.356	0.9554

**Table 5:** Comparison of the sorption properties of various sorbents towards RB5 and RY84 dyes.

DYE	Sorbent	Sorption Capacity [mg/g]	pH of Sorption	Duration of Sorption [min]	Source
RB5	Chitosan flakes DD = 90%	451.5	4	720	[11]
	Activated carbon Filtrasorb 400 (commercial)	198.0	5.2	400	[44]
	Aminated rapeseed husks (activated with epichlorohydrin)	135.8	3	120	This work
	Chitin flakes from snow crab shells (prod. by BioLog Heppe)	131.6	3	360	[45]
	Activated carbon (powder)	125.8	2	240	[10]
	Aminated wheat straw (activated with epichlorohydrin)	91.0	3	210	[40]
	Aminated buckwheat hulls (activated with epichlorohydrin)	85.2	3	300	[19]
	Aminated goldenrod biomass (activated with epichlorohydrin)	71.3	3	120	[39]
	Activated carbon modified with SPC	69.9	2	<60	[46]
	Activated carbon (powdered)	58.8	-	-	[47]
	Aminated sunflower seed shells (activated with epichlorohydrin)	51.0	3	240	[34]
	Activated carbon from bamboo	39.0	2	60	[38]
	Activated carbon from Carob tree	36.9	2	120	[42]
	Biochar from gasification residues	35.7	-	90	[43]
	Rape stalks (waste)	32.8	2.5	30	[48]
	Banana peel (powder)	26.9	3	60	[49]
	Activated carbon from palm shell	25.1	2	300	[37]
	Wood (walnut) activated carbon	19.3	5	400	[50]
	Wheat straw	15.7	7	195	[40]
	Rapeseed husks	15.2	3	180	This work
	Beech sawdust	13.9	3	1440	[16]
	Seed scales of *Eriobotrya japonica*	13.8	3	150	[17]
	Cotton seed husks	12.9	2	30	[51]
	Buckwheat hulls	4.43	3	300	[19]
	Sunflower seed shells	2.9	3	210	[34]
	Cotton fibers	2.7	3	240	[36]
	Goldenrod biomass	2.3	3	150	[39]
	Macadamia seed husks	1.2	3	510	[52]
	Sunflower biomass	1.1	2	210	[41]
	Pumpkin seed husks	1.0	3	60	[35]
RY84	Aminated rapeseed husks (activated with epichlorohydrin)	114.2	3	120	This work
	Aminated sunflower seed hulls (activated with epichlorohydrin)	63.3	3	240	[34]
	Aminated goldenrod biomass (activated with epichlorohydrin)	59.3	3	120	[39]
	Aminated cotton fibers (activated with epichlorohydrin)	43.3	2	240	[36]
	Activated carbon from the *Borassus flabellifer* plant	40.0	-	-	[53]
	Cotton fibers	15.9	2	240	[36]
	Rapeseed husks	13.7	3	180	This work
	Wool	11.0	7	180	[54]
	Sunflower seed husks	4.2	2	90	[34]
	Goldenrod biomass	2.3	3	180	[39]

## Data Availability

The data presented in this study are available on request from the corresponding author.

## Supplementary Materials

The following supporting information can be downloaded at: https://www.mdpi.com/article/10.3390/molecules29040843/s1, Table S1: Characteristics of dyes used in the study.
