# Supplementary material for: Aminated Rapeseed Husks (Brassica napus) as an Effective Sorbent for Removing Anionic Dyes from Aqueous Solutions
Source: Molecules. 2024 Feb 14;29(4):843. doi: 10.3390/molecules29040843 (PMC10892382; doi:10.3390/molecules29040843)
Supplement: Supplementary file 1 [file molecules-29-00843-s001.zip › molecules-2824947-supplementary.pdf]

Supplementary materials:

Table S1. Characteristics of dyes used in the study

Dye Name	Reactive Black 5 (RB5)	Reactive Yellow 84 (RY84)
Structural formula	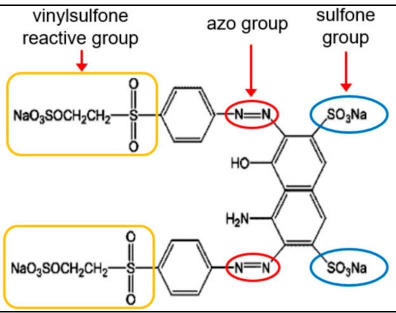	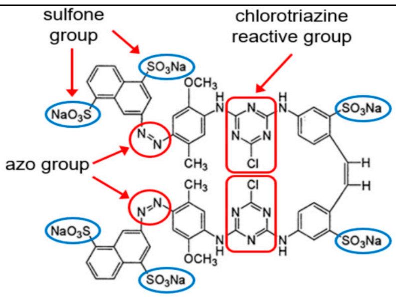
Chemical formula	$C_{26}H_{21}N_5Na_4O_{19}S_6$	$C_{56}H_{38}Cl_2N_{14}Na_6O_{20}S_6$
Molecular weight	991 g/mol	1628 g/mol
Dye class	double azo dye	double azo dye
Dye type	anionic (reactive)	anionic (reactive)
Type of reactive groups	vinylsulfone	chlorotriazine
λ_{max}	598 nm	356 nm
Uses	dyeing wool, polyamide fiber, cotton viscose	dyeing cotton, silk, polyester
Hazards	May be able to cause mutations, may be associated with the development of bladder cancer	May cause sensitization by inhalation; may be able to cause mutations
Other trade names	Begazol Black B; Remazol Black B	Active Yellow HE-4R; Lamafix Yellow HER.
